# Urban-rural disparity in the utilization of national community-based hypertension monitoring service—results from the China Health and Retirement Longitudinal Study, 2015

**DOI:** 10.7717/peerj.7842

**Published:** 2019-10-15

**Authors:** Hongxun Song, Da Feng, Ruoxi Wang, Shangfeng Tang, Bishwajit Ghose, Gang Li, Xiaoyu Chen, Zhanchun Feng

**Affiliations:** 1 School of Medicine and Health Management, Tongji Medical College, Huazhong University of Science & Technology, Wuhan, China; 2 School of Pharmacy, Tongji Medical College, Huazhong University of Science & Technology, Wuhan, China; 3 School of International Development and Global Studies, University of Ottawa, Ottawa, Canada

**Keywords:** Service utilisation, Urban, Rural, China, Equity, Hypertension monitor

## Abstract

**Background:**

Since 2009, community-based hypertension monitoring service (CBHMS) has been provided free of charge by the Chinese government as part of the national Essential Public Health Services (EPHS) policy. This study aimed to examine the disparity in the utilization of CBHMS between urban and rural community-dwelling middle-aged and older adults with hypertension.

**Methods:**

Subjects were 3,479 community-residing hypertensive patients, identified from the China Health and Retirement Longitudinal Study (CHARLS), 2015, a nationally representative survey of Chinese residents aged 45 years and older. The utilization of CBHMS was defined as having one’s blood pressure (BP) examined at least once a season by community or village doctors. Rates of CBHMS use of urban and rural residents with hypertension were compared by using chi-square test. Multiple logistic regression analyses were conducted to examine factors associated with the utilization of CBHMS of hypertensive patients.

**Results:**

CBHMS was significantly more likely to be used by rural than urban middle-aged and older residents with hypertension (38.6% vs. 25.1%, *P* < 0.001). Results from multiple logistic regression analyses showed that urban patients who were living in central (OR = 0.37) and western (OR = 0.48) regions (vs. eastern region), had an educational attainment of middle school (OR = 0.33) and college and above (OR = 0.48) (vs. illiterate), and were not taking antihypertensive agents (OR = 0.26) were less likely to use CBHMS, while rural patients who had no medical insurance (OR = 0.56), and were not taking antihypertensive agents (OR = 0.31) were less likely to use CBHMS.

**Conclusions:**

The national CBHMS is more likely to be used by rural middle-aged and older adults with hypertension in China. The urban-rural difference in the utilization of CBHMS may be resulted from the different demographics of urban and rural middle-aged and older residents and uneven distributions of health services resources between urban and rural areas. Urban-rural disparities in characteristics of CBHMS use should be taken into consideration when promoting the utilization of CBHMS in China.

## Introduction

Since the incidence of hypertension rises with age, the prevalence of hypertension is increasing in China partly because of the rapid growth of the elderly population (Chen et al., 2015; Feng, Pang & Beard, 2014; Gordon-Larsen et al., 2018). According to a recent study, the prevalence of hypertension in China is 44.7% among people aged 35–75 years, of whom 92.6% are 45 years and older (Lu et al., 2017). Hypertension has been associated with a higher risk of mortality caused by cardiovascular diseases (CVD), accounting for about one-third of the deaths in China (Lewington et al., 2016). As the main target of intervention to reduce CVD risk (Bi et al., 2015; Li, Zhao & Hu, 2016; Yan et al., 2014), hypertensive patients are encouraged to have their blood pressure (BP) monitored regularly, because this can promote the use of medications and healthy lifestyle behaviors (Zhang, Wang & Joo, 2017). Community-based hypertension monitoring for hypertensive patients has been proved to be effective for hypertension control in China as well as in other countries (Wang & Hoy, 2003; Zhang, Wang & Joo, 2017; Zhou et al., 2013).

In 2009, a national program, namely “the Essential Public Health Services (EPHS)”, was launched as part of the New Health Care System Reform in China (Zhou et al., 2013). The EPHS specially defines screening and monitoring of chronic diseases as basic healthcare service for community-dwelling patients. This service should be provided free of charge. In China, primary care institutions such as community health centers in urban areas and township hospitals in rural areas are responsible for the provision of free health examinations for all hypertensive patients livings in their catchment areas, including BP measurement once a season. Based on the results of hypertension monitoring, primary care physicians (PCPs) are required to give instructions on medication treatment and healthy lifestyle (Tang et al., 2015). Evidence from empirical studies has shown that community-based hypertension monitoring service (CBHMS) in community can help patients achieve a better BP control outcome (Ma, 2017).

To improve the quality of EPHS in China, it is necessary to have national data on the utilization of EPHS including CBHMS. However, up-to-date national data on the use of CBHMS are still very limited. A previous national study reported that, by 2013, 8.1% Chinese hypertensive patients had their blood pressure checked at primary care settings at least once during the 12 months prior to the study (Zhang et al., 2018). Nevertheless, this study defined hypertension monitoring in a loose way, blood pressure checked by PCPs once a year, not quarterly as defined by the guideline of China’s EPHS protocol (Song et al., 2019).

In the context of rapid social changes in China in recent decades, increasing disparities in rural–urban health services utilization have been recognized as a major source of health inequity in contemporary China. It is reported that rural residents used physician services more than urban residents (Liu et al., 2007). The uneven development between urban and rural areas would result in the urban-rural health inequity, including health services resource allocation (Dai, 2015), which in turn may contributes to the disparity in the use of CBHMS. For example, the urban health insurance system has a larger and more comprehensive coverage than rural medical insurance system, which would affect access to health service (Fang et al., 2019). However, few empirical studies have examined the urban-rural disparity in health service utilization among hypertensive patients in China. Estimating the urban-rural disparity in utilization of CBHMS can benefit the development and implementation of health policy. Accordingly, this study aimed to investigate the urban-rural disparities in the utilization of CBHMS among hypertensive adults at the national level, as well as factors associated with the use of it.

## Materials & Methods

### Data and sample

Data for this study came from the 2015 China Health and Retirement Longitudinal Study (CHARLS), which contained information about community-based hypertension management. CHARLS adopted a four-stage stratified cluster sampling to recruit participants. In the first stage, 150 county-level units from 28 provinces were selected to provide a mix of urban and rural settings, with a wide variation in the level of economic development. The second stage randomly chose 939 primary sampling units (PSUs) (470 villages and 469 communities) from the above county-level units. All the dwellings in each selected PSU were outlined on Google Earth maps using the CHARLS-GIS software, which was specifically designed for the study. The third stage randomly selected 24 mapped households from each PSU (Zhao, John & Yang, 2013). The last stage randomly selected 1 adult aged 45 years and older from each household. Finally, a total of 21,097 respondents were successfully enrolled in 2015. Of these respondents, 15,247 completed the blood pressure test, 5,884 were identified as having hypertension with 3,479 having information about community-based BP monitoring. The current analysis focused on the 3,479 residents with hypertension.

The original CHARLS was approved by the Ethical Review Committee at Peking University (IRB00001052–11015). A “Letter to the Residents” leaflet was sent to each of the selected households. All participants provided written informed consent before the household survey. This study’s datasets are publicly available at http://charls.pku.edu.cn/en/page/data/2015-charls-wave4.

### Measurement

The study sample were hypertensive patients who had undergone BP measurements in CHARLS. Before testing BP, respondents were asked to relax and remain seated while their BP was measured thrice at 5-minute intervals during the daytime, by using an Omron HEM-7200 Monitor. Medication treatment and other information were collected based on face-to-face interviews. Hypertension was defined as: (i) average systolic BP ≥ 140 mmHg or average diastolic BP ≥ 90 mmHg, or (ii) the respondent was currently taking antihypertensive agents (either chemical antihypertensive drugs or traditional Chinese herbal products).

All patients were asked: “How often did you have your blood pressure examined by community/village doctors?”. Answers for this question included “No”, “Once a week”, “Twice a month”, “Once a month”, “Once every two months”, “Once every season”, “Once half a year”, “Once every year”. Respondents who reported once every season or more were categorized as users of CBHMS.

Respondents’ socio-demographic variables included age (45–59, 60–69, >70 years), sex, educational attainment (illiterate, primary school, junior middle school, and college and above), annual household income, marital status (married vs. unmarried), residence place (rural vs. urban) and having medical insurance.

### Statistical analysis

Chi-square bivariate tests were used to compare rates of utilization of CBHMS of urban and rural residents. Multiple logistic regression analysis was used to identify factors significantly associated with the utilization of CBHMS, which entered CBHMS (yes/no) as the dependent variable, and all socio-demographic and clinical variables simultaneously as independent variables. We repeated this analysis with the sample of urban and rural hypertensive patients to identify factors associated with use of CBHMS among urban and rural patients, respectively. Odds ratio (OR) and their corresponding 95% confidence intervals (CIs) were used to quantify the association between factors and CBHMS use. The statistical significance of test level was set at 0.05 (two-tailed). SPSS 13.0 software (SPSS Inc., Chicago, USA) was used for all the analysis.

## Results

### Sample characteristics

As shown in Table 1, among the total 3,479 hypertensive subjects, 67.7% were older than 60 years and the mean age was 64.2 years (SD = 9.52, range 45–102). About 26.0% of subjects had no formal schooling. Most subjects were married. About half were living in rural areas. The rate of patients taking antihypertensive agents was 88.1%. Urban subjects had significantly higher level of education than rural subjects (*P* <  0.001).

**Table 1:** Basic characteristics of the study population (*n* = 3, 479).

Variable	Category	**Total** N(%)	**Urban** N(%)	**Rural** N(%)
Region	Western	987 (25.30)	388 (21.29)	599 (29.46)
	Central	843 (34.14)	362 (33.80)	481 (34.48)
	Eastern	1649 (40.57)	713 (44.91)	936 (36.06)
Marital status	Married	2909 (83.05)	1239 (80.69)	1670 (85.32)
Sex	Male	1592 (46.15)	655 (45.28)	937 (47.06)
Age level	45–59	1152 (32.27)	463 (31.52)	689 (33.04)
	60–69	1366 (38.65)	588 (39.68)	778 (37.58)
	70–	961 (29.08)	412 (28.80)	549 (29.37)
Household income*	Poor	870 (22.56)	321 (18.38)	549 (26.90)
	Near poor	870 (23.17)	260 (16.70)	610 (29.87)
	Middle income	869 (28.51)	425 (33.94)	444 (22.87)
	High income	870 (25.76)	457 (30.98)	413 (20.36)
Education	Uneducated	977 (25.97)	272 (16.88)	705 (35.39)
	Primary school	1523 (42.70)	637 (41.36)	886 (44.09)
	Junior middle school	632 (19.27)	313 (23.05)	319 (15.35)
	High school and above	347 (12.07)	241 (18.72)	106 (5.17)
Having medical insurance	Yes	3249 (93.58)	1368 (94.22)	1181 (92.91)
Taking medicine	Yes	3090 (88.23)	1296 (87.73)	1794 (88.55)

**Notes.**

a Household income were categorized into 4 groups based on the 25th, 50th, and 75th percentiles-poor (0–1,305 RMB), near poor (1,305–16,490 RMB), middle income (16,490-49,595 RMB), and rich (above 49,595 RMB).

### Utilization of CBHMS

Overall, only 33.2% of the subjects had their BP monitored by PCPs. The utilization rates were significantly lower in urban than rural patients (25.1% vs. 38.6%, *χ*2 = 56.43, *P* < 0.001).

Results from the chi-square test showed that urban patients who resided in western and central China (vs. eastern China), were men, had no medical insurance, and did not take antihypertensive agents were less likely to use CBHMS, while rural patients who had no medical insurance and did not take antihypertensive agents were less likely to use CBHMS (Table 2).

**Table 2:** Percentage of patients who reported use of community-based hypertension monitoring service.

Variable	Category	Urban (*N* = 1,473)			Rural (*N* = 2,025)		
		N (%)	*χ*	*p*	N (%)	*χ*	*p*
Region	Western	81 (20.87)			227 (37.89)		
	Central	67 (18.50)			167 (34.71)		
	Eastern	238 (33.38)			379 (40.49)		
			**37.63**	<**0.001**		8.164	0.097
Marital status	Married	318 (25.66)			630 (37.72)		
	Unmarried	68 (29.05)			143 (40.28)		
			1.42	0.339		0.72	0.427
Sex	Male	147 (22.44)			336 (35.85)		
		239 (29.21)			337 (30.97)		
			**12.88**	**0.002**		2.113	0.161
Age	45–59	119 (25.70)			262 (38.02)		
	60–69	151 (25.68)			308 (39.58)		
	70–	116 (28.15)			203 (36.97)		
			5.09	0.367		2.21	0.354
Household income*	Poor	92 (28.66)			202 (36.79)		
	Near poor	79 (30.38)			249 (40.81)		
	Middle income	107 (25.17)			163 (36.71)		
	High income	108 (23.63)			159 (38.49)		
			6.14	0.214		1.74	0.658
Education	Uneducated	93 (34.19)			287 (40.70)		
	Primary school	187 (29.35)			324 (36.56)		
	Junior middle school	63 (20.12)			116 (36.36)		
	High school and above	43 (17.84)			46 (43.39)		
			**32.49**	**0.025**		2.63	0.429
Having medical insurance	Yes	363 (26.53)			736 (62.32)		
	No	23 (21.90)			37 (4.383)		
			0.49	0.515		**7.73**	**0.009**
Taking Medicine	Yes	365 (28.16)			724 (40.35)		
	No	21 (11.86)			49 (21.21)		
			**25.90**	<**0.001**		**45.68**	<**0.001**

**Notes.**

a Household income were categorized into four groups based on the 25th, 50th, and 75th percentiles-poor (0–1,305 RMB), near poor (1,305–16,490 RMB), middle income (16,490-49,595 RMB), and rich (above 49,595 RMB).

### Factors associated with the utilization of CBHMS

Table 3 shows results on factors associated with utilization of CBHMS, stratified by residence place. After adjusting for socio-demographic variables, urban patients were still significantly less likely to use this service than their rural counterparts (OR = 0.57, 95% CI [0.45–0.73], *P* < 0.001). Urban patients who were living in central (OR = 0.37, 95% CI [0.23–0.57], *P* < 0.001) and western (OR = 0.48, 95% CI [0.30–0.78], *P* < 0.001) regions (vs. eastern region), had an educational attainment of middle school (OR = 0.33, 95% CI [0.21–0.57], *P* < 0.001) and college and above (OR = 0.48, 95% CI [0.24–0.97], *P* < 0.01) (vs. illiterate), and were not taking antihypertensive agents (OR = 0.26, 95% CI [0.12–0.56], *P* < 0.001) were less likely to use CBHMS. Rural patients who had no medical insurance (OR = 0.56, 95% CI [0.35–0.88], *P* = 0.012), and were not taking antihypertensive agents (OR = 0.31, 95% CI [0.21–0.45], *P* <  0.001) were less likely to use CBHMS.

**Table 3:** Factors associated with utilisation of community-based hypertension monitoring.

Variable	Category	Total OR (95% CI)	Urban OR (90% CI)	Rural OR (90% CI)
Urbanity	Rural	1	**–**	**–**
	Urban	0.574^**^	**–**	**–**
		(0.451 to 0.732)	**–**	**–**
Region	Eastern	1	1	1
	Western	0.674^**^	0.483^**^	0.862
		(0.517 to 0.878)	(0.298 to 0.782)	(0.626 to 1.185)
	Central	0.532^**^	0.366^**^	0.713
		(0.406 to 0.697)	(0.233 to 0.576)	(0.516 to 0.984)
Marital status	Unmarried	1	1	1
	Married	0.812	0.918	0.770
		(0.632 to 1.043)	(0.582 to 1.448)	(0.580 to 1.023)
Sex	Female	1	1	1
	Male	0.899	0.779	0.963
		(0.751 to 1.076)	(0.578 to 1.051)	(0.775 to 1.196)
Age	45–59	1	1	1
	60–69	0.802	0.692	0.965
		(0.601 to 1.069)	(0.395 to 1.212)	(0.757 to 1.229)
	70–	0.800	0.849	0.802
		(0.598 to 1.071)	(0.509 to 1.417)	(0.600 to 1.073)
Household income	High income	1	1	1
	Poor	1.021	1.160	0.914
		(0.791 to 1.317)	(0.761 to 1.768)	(0.684 to 1.122)
	Near poor	1.150	1.331	0.985
		(0.862 to 0.860)	(0.779 to 2.273)	(0.747 to 1.300)
	Middle income	0.873	0.845	0.852
		(0.680 to 1.122)	(0.562 to 1.271)	(0.631 to 1.149)
Education	Uneducated	1	1	1
	Primary school	0.876	0.807	0.833
		(0.692 to 1.108)	(0.510 to 1.278)	(0.654 to 1.061)
	Junior middle school	0.568^**^	0.328^**^	0.842
		(0.424 to 0.761)	(0.213 to 0.571)	(0.596 to 1.189)
	College and above	0.676	0.478^**^	0.937
		(0.412 to 1.108)	(0.236 to 0.969)	(0.695 to 1.724)
Having medical insurance	Yes	1	1	1
	No	0.608^*^	0.718	0.558^*^
		(0.419 to 0.883)	(0.381 to 1.353)	(0.354 to 0.878)
Taking Medicine	Yes	1	1	1
	No	0.296^**^	0.263^**^	0.305^**^
		(0.205 to 0.427)	(0.123 to 0.560)	(0.206 to 0.451)

**Notes.**

OR = Odds Ratio.

95% CI = 95% Confidence interval for odds ratio.

* *P* < 0.05.

** *P* < 0.01.

## Discussion

Based on nationally representative data from CHARLS, this study examined urban-rural difference in the utilization of CBHMS and its associated factors among Chinese adults aged 45 years or older. The rate of use of CBHMS was 33.2% in China in 2015. This service was more frequently used by rural than urban community-dwelling patients. Utilization of the service in rural was negatively associated with no medical insurance, while in urban it was negatively associated with higher level of education and residing in central and western areas of China.

To date, few studies have examined the utilization of CBHMS in China. In Germany, a community-based research reported that only 33.7% of the hypertensive patients had received at least two BP examinations in 2016 (Jacob, Seitz & Kostev, 2018). Compared to this estimate, we observed a relatively high rate of utilization of CBHMS, because our definition of utilization is more strict than that of the Germany study. However, the figure that only one-third of the Chinese hypertensive persons regularly had their BP measured (once a season at least) by community or village doctors still indicates that the CBHMS was underutilized. There may be several reasons for the underutilization. First, as shown in this study, about a half of the hypertensive patients were undiagnosed. Many patients did not use the service since they were unaware of it (Feng, Pang & Beard, 2014). Second, the shortage of primary care providers in Chinese communities may also limit the delivery of serve for hypertension management, including BP examinations or counselling (Zhang et al., 2018). Third, in China, a tiered diagnosis and treatment system has not been well-established so some patients may directly receive the service at large general hospitals (Liu et al., 2018; Yip & Hsiao, 2014). In general, due to the relatively limited quality of primary health care, many Chinese hypertensive patients are not willing to seek management service from community or village doctors (Sun et al., 2019).

Although the access to CBHMS should be universal and equal, the allocation of health resources is uneven between urban and rural areas and the provision of healthcare service is inadequate in rural areas in China. Because of this, the central government of China has devoted more resources for managing chronic diseases in underdeveloped areas in recent years (Tian et al., 2013). Accordingly, a larger improvement of BP monitoring was observed in western regions of China with relatively low economic status and a large proportion of rural population (Zhang et al., 2018). On the other hand, because primary care services have been widely available in rural areas of China and large Chinese general hospitals are mainly located in distant large cities, rural patients are more likely to use CBHMS (Kawazoe et al., 2018; Zhong et al., 2019).

This study also found different factors associated with CBHMS between urban and rural areas, which may reflect the significant cultural, social, and economic differences between urban and rural China. The results replicating the finding that education was related to blood pressure measurement among urban elderly (Wang et al., 2018). In general, urban residents have a higher level of education than rural individuals. Individuals with a higher level of education may have more opportunities to use home sphygmomanometer and to visit physicians of larger hospitals for managing hypertension (Pandit et al., 2009; Zahid et al., 2017). Illiterate patients often have a low socio-economic status and are more likely to have their BP checked by the community doctors free of charge (Akpolat et al., 2012; Feng, Pang & Beard, 2014). This study showed regional disparities in the utilization of CBHMS among eastern, western, and central China in urban areas only. The utilization rate was higher in eastern urban areas, which could be ascribed to high service quality of grassroots health provision and sufficient financial input for health services relative to other areas (Zhou et al., 2017). Due to the low economic status of rural residents in China, rural patients with medical insurance were more likely to use CBHMS because this health service can be paid by the medical insurance scheme, not out of pocket.

This study also showed that medication treatment was significantly associated with CBHMS use. Because hypertension monitoring can improve medication compliance, which in turn results in medication treatment, this finding may suggest that CBHMS use is the cause of medication treatment. The other possible explanation is that patients taking antihypertensive drugs are those who care for their health; therefore, they have the motivation to measure BP (Wang et al., 2014).

This study has several limitations. First, medication and use of community BP examination were collected via interview, not verified by reviewing the medical records. Our findings may be subject to recall bias. Second, the survey focused on patients with hypertension; whereas arguably, collecting both patients and service providers’ information could generate a more comprehensive picture regarding the characteristics of service utilization. Finally, due to the cross-sectional research design, the causality of BP monitoring and its associated factors cannot be ascertained.

## Conclusions

The national CBHMS is more likely to be used in rural areas. Disparities in utilization of urban and rural services still exist. The urban-rural difference in the utilization of CBHMS may be resulted from the different demographics of urban and rural middle-aged and older residents and uneven distributions of health services resources between urban and rural areas. Factors associated with the utilization of CBHMS are also different between urban and rural middle-aged and elderly hypertensive patients. Further implementation of CBHMS in China should take urban-rural disparity into consideration.

##  Supplemental Information

10.7717/peerj.7842/supp-1 Supplemental Information 1 The urban-rural disparity still existed among education, insurance, cohabitation status and different regions

10.7717/peerj.7842/supp-2 Supplemental Information 2 Complex sampling plan was created according to stratification, cluster and weights of this survey

## Additional Information and Declarations

### Data Availability

The following information was supplied regarding data availability: The raw measurements are available in the Supplemental Files.
